# Complete mitochondrial genome of *Thuja sutchuenensis* and its implications on evolutionary analysis of complex mitogenome architecture in Cupressaceae

**DOI:** 10.1186/s12870-023-04054-9

**Published:** 2023-02-07

**Authors:** Changying Xia, Jingling Li, Youwei Zuo, Ping He, Huan Zhang, Xiaoxia Zhang, Binru Wang, Jiabin Zhang, Jie Yu, Hongping Deng

**Affiliations:** 1 Center for Biodiversity Conservation and Utilization, School of Life Sciences, Southwest University, 400715 Chongqing, China; 2 Low Carbon and Ecological Environment Protection Research Center, Chongqing Academy of Science and Technology, 400715 Chongqing, China; 3 College of Horticulture and Landscape Architecture, Southwest University, 400715 Chongqing, China; 4 State Key Laboratory of Systematic and Evolutionary Botany , Institute of Botany Chinese Academy of Sciences, 100093 Beijing, China

**Keywords:** *Thuja sutchuenensis*, Mitogenome, Gymnosperms, Repeat sequence, Genetic evolution

## Abstract

**Background:**

The complex physical structure and abundant repeat sequences make it difficult to assemble the mitogenomes of seed plants, especially gymnosperms. Only approximately 33 mitogenomes of gymnosperms have been reported. However, as the most widely distributed and the second largest family among gymnosperms, Cupressaceae has only six assembled mitogenomes, including five draft mitogenomes and one complete mitogenome, which has greatly hindered the understanding of mitogenome evolution within this large family, even gymnosperms.

**Results:**

In this study, we assembled and validated the complete mitogenome of *Thuja sutchuenensis*, with a size of 2.4 Mb. Multiple sequence units constituted its complex structure, which can be reduced to three linear contigs and one small circular contig. The analysis of repeat sequences indicated that the numbers of simple sequence repeats increased during the evolutionary history of gymnosperms, and the mitogenome of *Thuja sutchuenensis* harboured abundant extra-long repeats (more than 5 kb). Additionally, the longest repeat sequence identified in these seven gymnosperms also came from the mitogenome of *Thuja sutchuenensis*, with a length of up to 47 kb. The analysis of colinear blocks and gene clusters both revealed that the orders of mitochondrial genes within gymnosperms was not conserved. The comparative analysis showed that only four tRNAs were shared by seven gymnosperms, namely, *trnD-GUC, trnE-UUC, trnI-CAU* and *trnY-GUA*. Furthermore, four genes have undergone potential positive selection in most gymnosperm species, namely, *atp8*, *ccmB*, *mttB* and *sdh4*.

**Conclusion:**

We successfully assembled the second complete mitogenome within Cupressaceae and verified that it consisted of multiple sequence units. Our study also indicated that abundant long repeats may contribute to the generation of the complex conformation of the mitogenome of *Thuja sutchuenensis*. The investigation of *Thuja sutchuenensis*’s mitogenome in our study provides new insight into further understanding the complex mitogenome architecture within gymnosperms.

**Supplementary Information:**

The online version contains supplementary material available at 10.1186/s12870-023-04054-9.

## Background

Due to the complicated physical structure, sequence composition, and abundance of repeat sequences, there are still certain challenges associated with the assembly and annotation of vascular plant mitochondrial genomes [1–5]. An increasing number of plant mitochondrial genomes are being reported as a result of the ongoing advancements in sequencing technology [5–8]. However, gymnosperms only make up a small portion of the published plant mitogenomes; the majority come from angiosperms. Only 33 gymnosperm mitogenomes (including draft mitogenomes) have been reported to date [4, 8–13]. Previous research has demonstrated that the gymnosperm mitogenome has undergone substantial changes in genomic size, physical structure, and gene composition [4, 8–13]. For example, the genome size of *Larix sibirica* Ledeb. was 33 times larger than that of *Ginkgo biloba* L. (*G. biloba*) [11, 12]. The related groups, *Pinus taeda* L. (*Pi. taeda*) and *Welwitschia mirabilis* Hook. f. (*W. mirabilis*), differ by 12 protein-coding genes (PCGs, 41 vs. 29) [11].

Gymnosperms can be classified into five main lineages, including Cycads, Ginkgo, Pinaceae, Gnetophytes, and Conifer II (non-Pinaceae conifers or Cupressophyta), according to recent studies [14]. Cupressaceae, as the most widely distributed and the second largest family among gymnosperms (the number of species is only second to Pinaceae), is an essential component of Conifer II. However, Cupressaceae has only reported six assembled mitogenomes to date, including five draft mitogenomes and one complete mitogenome [9, 10, 13]. This may greatly hinder a deep understanding of the evolution of mitogenomes in this large family, even gymnosperms.

*Thuja sutchuenensis* Franch. (*Th. sutchuenensis*), belonging to an astern Asian-North American disjunct genus, was first discovered by French missionary P. G. Farges in 1892 and officially published by Franche in 1899 [15, 16]. Since Farges collected it in 1900, this species has not been found in the wild for over 90 years [16]. As a result, it was listed as being "extinct in the wild" in some monographs and red lists [17–19]. *Th.* sutchuenensis was not rediscovered until 1999, during a comprehensive investigation of rare and endangered plants in Chengkou County [16]. However, recent studies have indicated that both the existing population and wild seedlings of *Th. sutchuenensis* were rare, and reproductive barriers were the main reason for its population decline [20–22]. Therefore, *Th. sutchuenensis* is now assessed as an endangered species and the I-class national key protected wild plant in China. Previous research has demonstrated that there is a close relationship between the mitogenome and cytoplasmic male sterility (CMS), and abortion occurred during the growth of ovulate strobilus, microstrobilus and seeds of *Th. sutchuenensis* [22–24]. Hence, studying the mitogenome of *Th. sutchuenensis* is helpful to understand the molecular mechanism of the reproductive barriers, and is of great significance for the protection of this endangered plant.

In this study, the complete mitogenome of *Th. sutchuenensis* was sequenced, assembled and validated. Then, we compared it with multiple reported mitogenomes of gymnosperms. Our research provides more evidence that the plant mitochondrial genome contains several sequence components with complex structures. The investigation of the *Th. sutchuenensis*' mitogenome also provides supporting data for the study of the reproductive obstacles of this endangered species and the genetic evolution of the gymnosperm mitogenome.

## Results

### Assembly, annotation and validation of *Th. sutchuenensis* mitochondrial genome

**Fig. 1:**
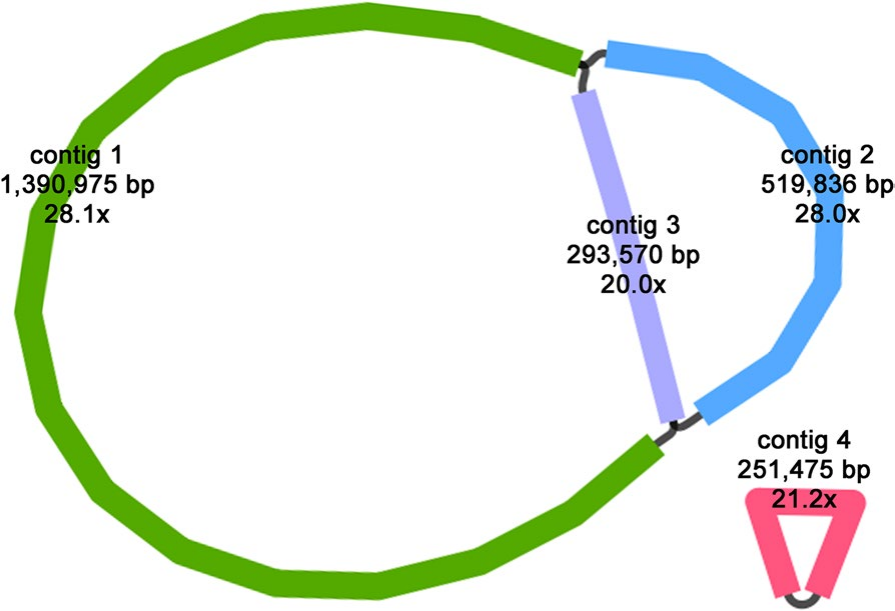
The assembly graph of the mitogenome of *Thuja sutchuenensis*

Assembly results indicated that the complete mitochondrial genome of *Th. sutchuenensis* is 2.46 Mb, including 4 contigs. Bandage [25] was used to visualize the complete mitogenome, which is composed of two components: a larger 2.2-Mb component (including 3 contigs, labelled 1–3) and a smaller 251-kb circular contig (labelled 4) (Fig. 1). Contigs 1, 2 and 3 had overlapping regions with each other, with lengths of 1,390,975 bp, 519,836 bp and 293,570 bp, respectively. These 3 contigs formed a multibranched structure and were treated as putative linear molecules here. The shortest segment, contig 4, was putatively circular with a length of 251,475 bp.

To validate the accuracy of the assembly conformation of *Th. sutchuenensis*, PCR and Sanger sequencing were carried out. We designed four pairs of specific primers (AF + AR, BF + BR, CF + CR and DF + DR) for PCR amplification. The two connected regions of contig 1 and contig 3 should successfully generate PCR products by primer pair AF + AR and BF + BR, respectively, while the connected regions of contig 1 and contig 2, contig 2 and contig 3 should successfully generate PCR products by primer pairs CF + CR and DF + DR, respectively. The PCR amplification results showed that the lengths of bands were consistent with that expected (Additional file 1: Fig. S1), and the Sanger sequencing results confirmed the presence of this complex connected structure of the assembly conformation (Additional file 1: Fig. S2).

**Fig. 2:**
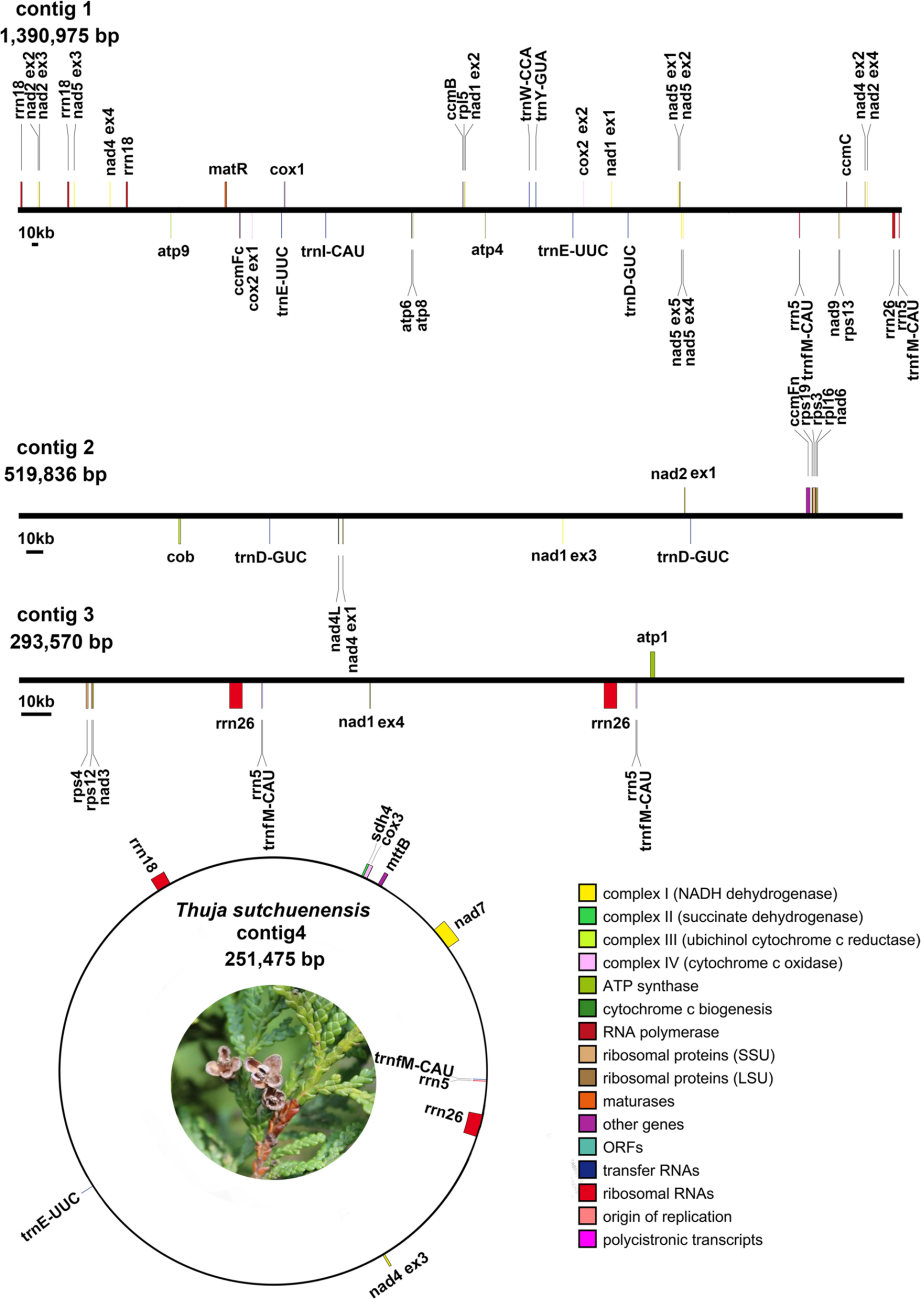
Gene map of the complete mitogenome of *Thuja sutchuenensis*. For contigs 1, 2, and 3, genes above and below the horizontal line are transcribed in clockwise and counterclockwise directions, respectively. For contig 4, genes inside and outside the outer circle are transcribed in clockwise and counterclockwise directions, respectively

According to the annotation results, the mitogenome of *Th. sutchuenensis* had 32 different PCGs with known functions, including 24 core mitochondrial genes and 8 variable genes. The core genes were composed of nine NADH dehydrogenase genes, five ATP synthase genes, four ubiquinol cytochrome c reductase genes, three cytochrome c oxidase genes, 1 transport membrane protein gene, 1 maturases gene and 1 cytochrome c biogenesis gene. The variable genes included five small subunits of ribosome genes (*rps*-), two large subunits of ribosome genes (*rpl*-) and one succinate dehydrogenase gene (Table 1). Fourteen introns were discovered in 5 of the 32 PCGs (*nad5* had four introns; *nad 1*, *nad 2* and *nad4* had three introns; and *cox2* had one intron). In addition, three rRNA genes, five tRNA genes of mitochondrial origin (*trnD-GUC, trnE-UUC, trnI-CAU, trnfM-CAU, trnY-GUA*), and one tRNA gene of chloroplast origin (*trnW-CCA*) were also annotated in the mitogenome. The relative order and direction of these genes are shown in the mitogenome map (Fig. 2).

**Table 1:** Gene composition in the mitogenome of *Thuja sutchuenensis*

Group of genes	Name of genes
ATP synthase	*atp1, atp4, atp6, atp8, atp9*
Cytochrome c biogenesis	*ccmB, ccmC, ccmFc, ccmFn*
Ubichinol cytochrome c reductase	*cob*
Cytochrome c oxidase	cox1, cox2^*^, cox3
Maturases	*matR*
Transport membrane protein	*mttB*
NADH dehydrogenase	nad1^*^, nad2^*^, nad3, nad4^*^, nad4L, nad5^*^, nad6, nad7, nad9
Large subunit of ribosome	*rpl5, rpl16*
Small subunit of ribosome	*rps3, rps4, rps12, rps13, rps19*
Succinate dehydrogenase	*sdh4*
Ribosomal RNAs	*rrn5, rrn18, rrn26*
Transfer RNAs of mitochondrial origin	*trnD-GUC, trnE-UUC, trnI-CAU, trnfM-CAU, trnY-GUA*
Transfer RNAs of chloroplast origin	*trnW-CCA*

Note: ‘*’ Labeled the genes that contain introns

### Repeat sequences in the mitogenomes of gymnosperms

**Fig. 3:**
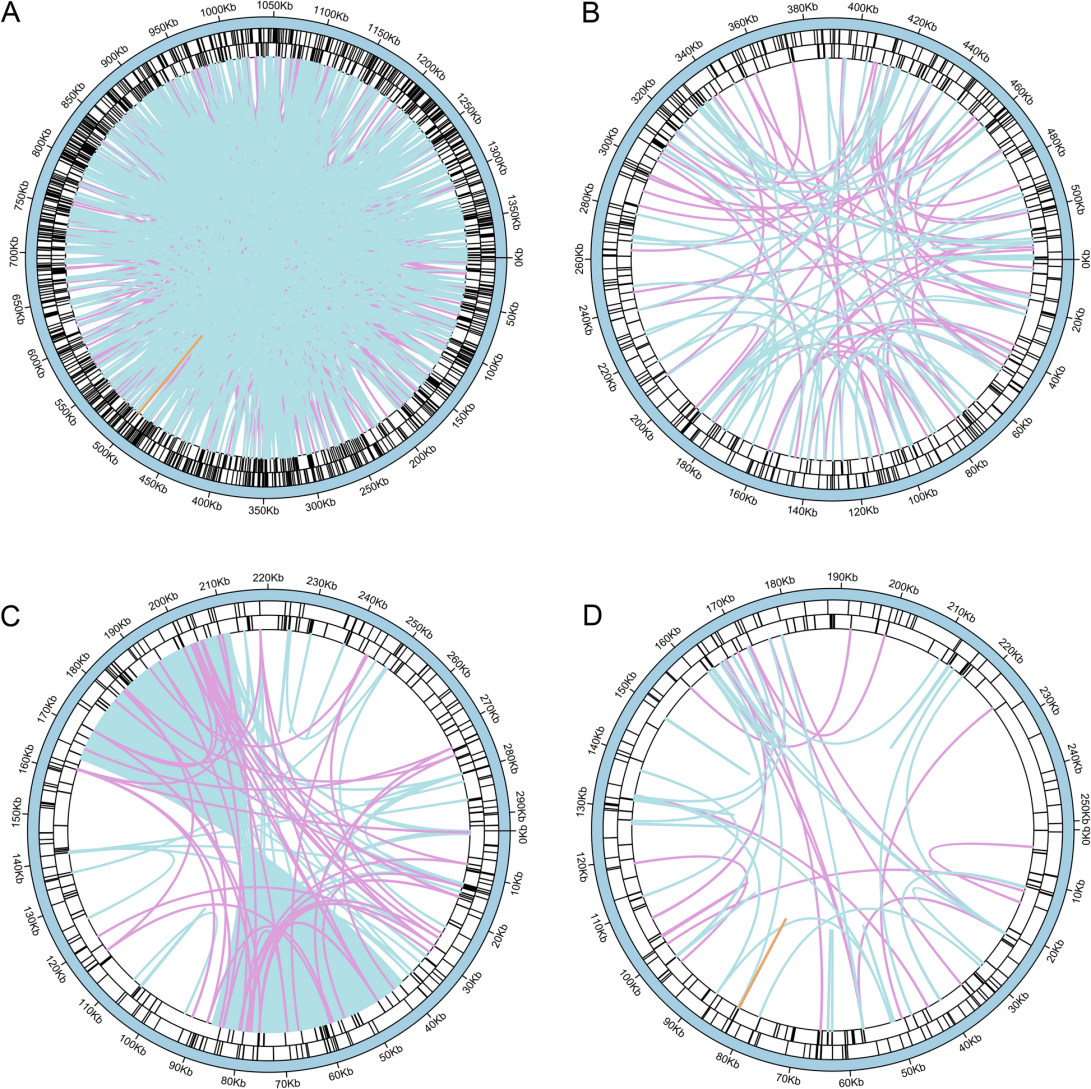
The distribution of repeats in the mitogenome of *Thuja sutchuenensis*. The inner circle shows the dispersed repeats with a length greater than or equal to 50 bp, in which blue represents forward repeats, purple represents palindromic repeats, orange represents reverse repeats. The two outer circles show tandem repeats and simple sequence repeats as short bars, respectively. **A, B, C** and **D** represent the distribution of repeat sequences of contig 1–4, respectively

The analysis of repeat sequences indicated that 894, 293, 202 and 127 simple sequence repeats (SSRs) were found in contigs1-4 of *Th. sutchuenensis*, respectively, and the tetranucleotide polymers accounted for the largest proportion in each contig (Additional file 1: Fig. S3, Additional file 2: Table S2.1-S2.4). In addition, more than 2,000 dispersed repeats with a length greater than or equal to 30 bp were detected in each contig (Additional file 2: Table S2.5-S2.8). Among these dispersed repeats, only three pairs of complementary repeats were observed in contig 2 and contig 4, respectively, but not in contig 1 and contig 3 (Additional file 1: Fig. S4). The analysis also identified abundant tandem repeats in these 4 contigs, with 769, 270, 181, and 116 tandem repeats found in contigs 1–4, respectively (Additional file 2: Table S2.9-S2.12). In total, 1,516 SSRs, 15,317 pairs of dispersed repeats, and 1,336 tandem repeats were found in the mitogenome of *Th. sutchuenensis*. Different types of repeat sequences are shown in Fig. 3 in a circle diagram. The analysis of long repeats (> 500 bp) showed that 31 long repeats were found in the whole mitogenome of *Th. sutchuenensis,* which were main located among contig1, contig 3, and contig 4 (Additional file 1: Fig. S5; Additional file 2: Table S2.13).

We also identified the repeat sequences of mitogenomes of six other representative gymnosperms and compared them with *Th. sutchuenensis*. The results indicated that all species had the highest proportion of tetranucleotide polymers except for *W. mirabilis* and *Taxus cuspidata* Sieb. et Zucc. (*Ta. cuspidata*)*,* which were dominated by pentanucleotide polymers and dinucleotide polymers, respectively (Additional file 1: Fig. S6A, Additional file 2: Table S2.14-S2.20). The total number of SSRs indicated that *Cycas taitungensis* C. F. Shen et al. (*C. taitungensis*) and *G. biloba* had the fewest SSRs (both less than 200), *Pi. taeda* and *W. mirabilis* had a moderate number of SSRs (500–700), while *Ta. cuspidata, Platycladus orientalis* (L.) Franco (*Pl. orientalis*) and *Th. sutchuenensis* harboured the most SSRs (both more than 1,000). Comparative analysis of dispersed repeats indicated that forward repeats and palindromic repeats accounted for the largest proportion in gymnosperms (Additional file 1: Fig. S6B, Additional file 2: Table S2.5-S2.8, S2.21-S2.27). Among the 7 gymnosperms, *Pl. orientalis* and *Th. sutchuenensis* harboured the largest number of extra-long repeats (more than 5,000 bp), far more than the other five gymnosperms (Additional file 1: Fig. S7; Additional file 2: Table S2.13, S2.21-S2.25, S2.28). And although the mitogenome of *Th. sutchuenensis* was slightly smaller than that of *Pl. orientalis*, it possessed the longest repeat sequence identified in seven gymnosperms, which was up to 47,016 bp long.

### Identification of mitochondrial plastid DNAs (MTPTs) and synteny analysis

The blast analysis for the mitogenome and plastid genome indicated that 17 homologous fragments were identified, with a total length of 3,481 bp, accounting for 0.14% of the total mitogenome (Additional file 1: Fig. S8, Table 2). Among those fragments, the longest was 660 bp, and the shortest was 63 bp. Annotation analysis for these homologous fragments indicated that only two incomplete rRNA genes (*rrn18* and *rrn26*) were found, which appeared in the mitochondrial and plastid genomes simultaneously (*rrn18/rrn16* and *rrn26/rrn23*). Therefore, this could not represent the existence of sequence migration between the two genomes, as these rRNA genes may be of a common origin. In conclusion, no significant sequence migration was detected between the chloroplast and mitochondrial genomes of *Th. sutchuenensis*.

**Table 2:** Mitochondrial plastid DNAs (MTPTs) identified in mitogenome of *Thuja sutchuenensis*

Fragments	Aligned length (bp)	Mitogenome		Plastome		Contained genes
		**Start**	**End**	**Start**	**End**	
mtpt1^1^	660	1365776	1366435	188	806	
mtpt2^1^	312	4909	5220	66118	66427	rrn18^*^
mtpt3^1^	312	78523	78834	66118	66427	rrn18^*^
mtpt4^1^	312	170693	171004	66118	66427	rrn18^*^
mtpt5^1^	138	5517	5654	65695	65832	rrn18^*^
mtpt6^1^	138	79131	79268	65695	65832	rrn18^*^
mtpt7^1^	138	171301	171438	65695	65832	rrn18^*^
mtpt8^1^	77	497,660	497736	41341	41418	
mtpt9^1^	90	1379228	1379317	62080	62171	rrn26^*^
mtpt10^1^	63	464043	464105	47178	47234	
mtpt11^2^	333	387365	387697	66340	66694	
mtpt12^2^	188	44446	44633	108914	109128	
mtpt13^3^	90	73197	73286	62080	62171	rrn26^*^
mtpt14^3^	90	197401	197490	62080	62171	rrn26^*^
mtpt15^4^	312	1782	2093	66118	66427	rrn18^*^
mtpt16^4^	138	2390	2527	65695	65832	rrn18^*^
mtpt17^4^	90	160683	160772	62080	62171	rrn26^*^

Note: ‘^1^’ labeled fragments located in mtDNA contig 1

‘^2^’ labeled fragments located in mtDNA contig 2

‘^3^’ labeled fragments located in mtDNA contig 3

‘^4^’ labeled fragments located in mtDNA contig 4

‘^*^’ labeled the genes that was incomplete

**Fig. 4:**
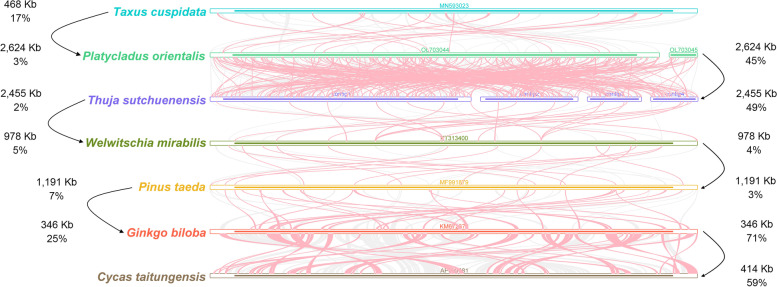
The collinear blocks among gymnosperm mitogenomes. Bars indicated the mitogenomes, and the ribbons showed the homologous sequences (> 500 bp) between the adjacent species. The red areas and gray areas indicate collinear blocks with inconsistent and consistent arrangement orders, respectively. The four lines of numbers beside the arrow represent the genome size of two species and the respective proportions of all homologous sequences between two species

Synteny analysis indicated that there were a large number (10,496) of homologous collinear blocks among the seven gymnosperms, especially between *Th. sutchuenensis* and *Pl. orientalis, C. taitungensis* and *G. biloba* (Fig. 4). The analysis also found that collinear blocks with inconsistent arrangement orders accounted for a large proportion (average proportion: 52%), which may indicate that these seven representative gymnosperms have experienced a large number of genome rearrangement events, and the mitogenome structure of gymnosperms was not conserved. Previous studies have identified 29 conserved gene clusters in angiosperms [26]. However, only 3 conserved gene clusters were found in the mitogenome of *Th. sutchuenensis*, namely, *cox3-sdh4*, *nad3-rps12* and *rps3-rpl16* (Additional file 1: Fig. S9). The number and composition of gene clusters varied greatly among these seven species, except for two species of Cupressaceae (*Th. sutchuenensis* and *Pl. orientalis*), which had three identical gene clusters.

### Variation in gene composition in the mitogenomes of gymnosperms

**Fig. 5:**
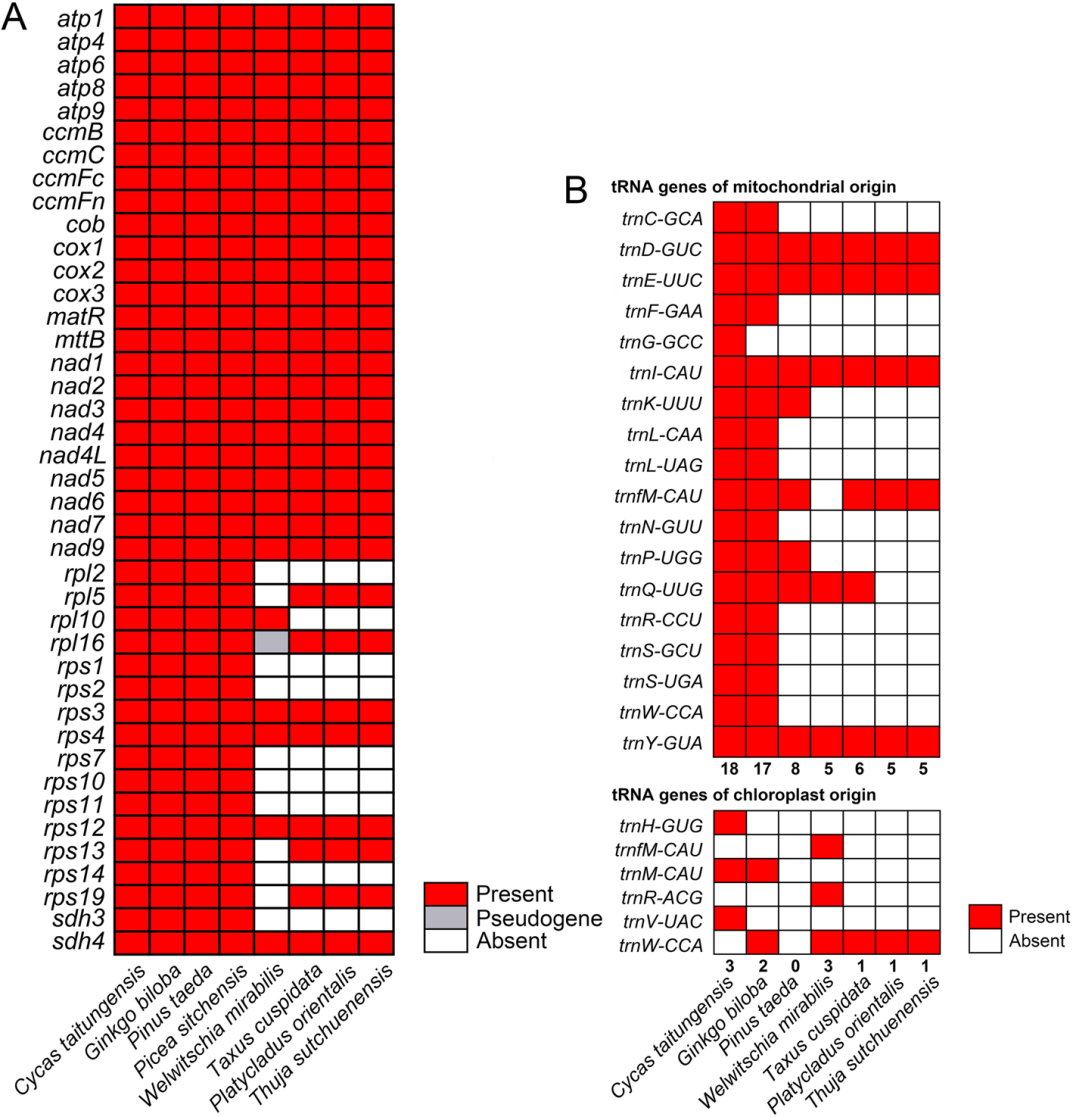
The composition of protein coding genes (**A**) and tRNA genes (**B**) in the 7 gymnosperm mitogenomes

We compared the mitogenomes of *Th. sutchuenensis* with 6 other representative gymnosperm species to show the different gene compositions among gymnosperm mitogenomes. Among the 7 species representing 5 lineages of gymnosperms, *C. taitungensis, G. biloba* and *Pi. taeda* had the highest number of PCGs (41), while the fewest PCGs were found in *W. mirabilis* (29, Fig. 5A, Additional file 1: Table S3.1). In addition, three species from Conifer II, including *Ta. cuspidata, Pl. orientalis* and *Th. sutchuenensis*, harboured identical numbers and compositions of PCGs (32). A comprehensive reannotation of tRNA for the mitogenomes of seven gymnosperms showed that compared with the basal group of gymnosperms (*C. taitungensis* and *G. biloba*), numerous loss events of mitochondrial-derived tRNA occurred in the evolutionary history of gymnosperms. Only four tRNAs were found to be conserved in seven gymnosperms, namely, *trnD-GUC, trnE-UUC, trnI-CAU and trnY-GUA* (Fig. 5B). Additionally, the occurrence of chloroplast-derived tRNAs seemed to be random. Interestingly, we found that *trnfM-CAU* (mt origin) was conserved in all species except for *W. mirabilis*, whereas *trnfM-CAU* (cp origin) was found only in this species.

### Variation in substitution rates of mitochondrial PCGs among gymnosperms

**Fig. 6:**
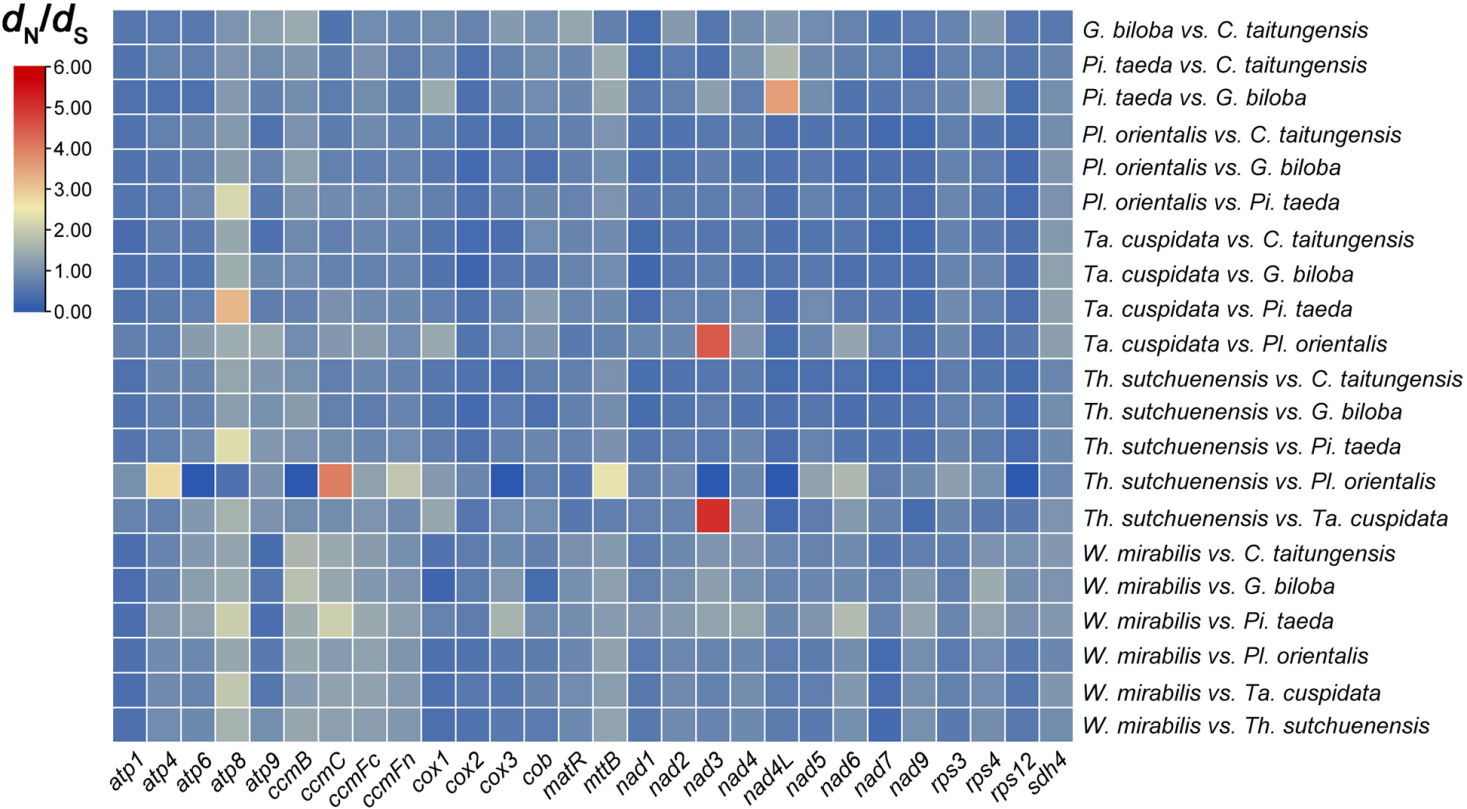
Heatmap of pairwise *d*_N_/*d*_S_ ratios among 28 mitochondrial genes in the 7 gymnosperm mitogenomes

**Fig. 7:**
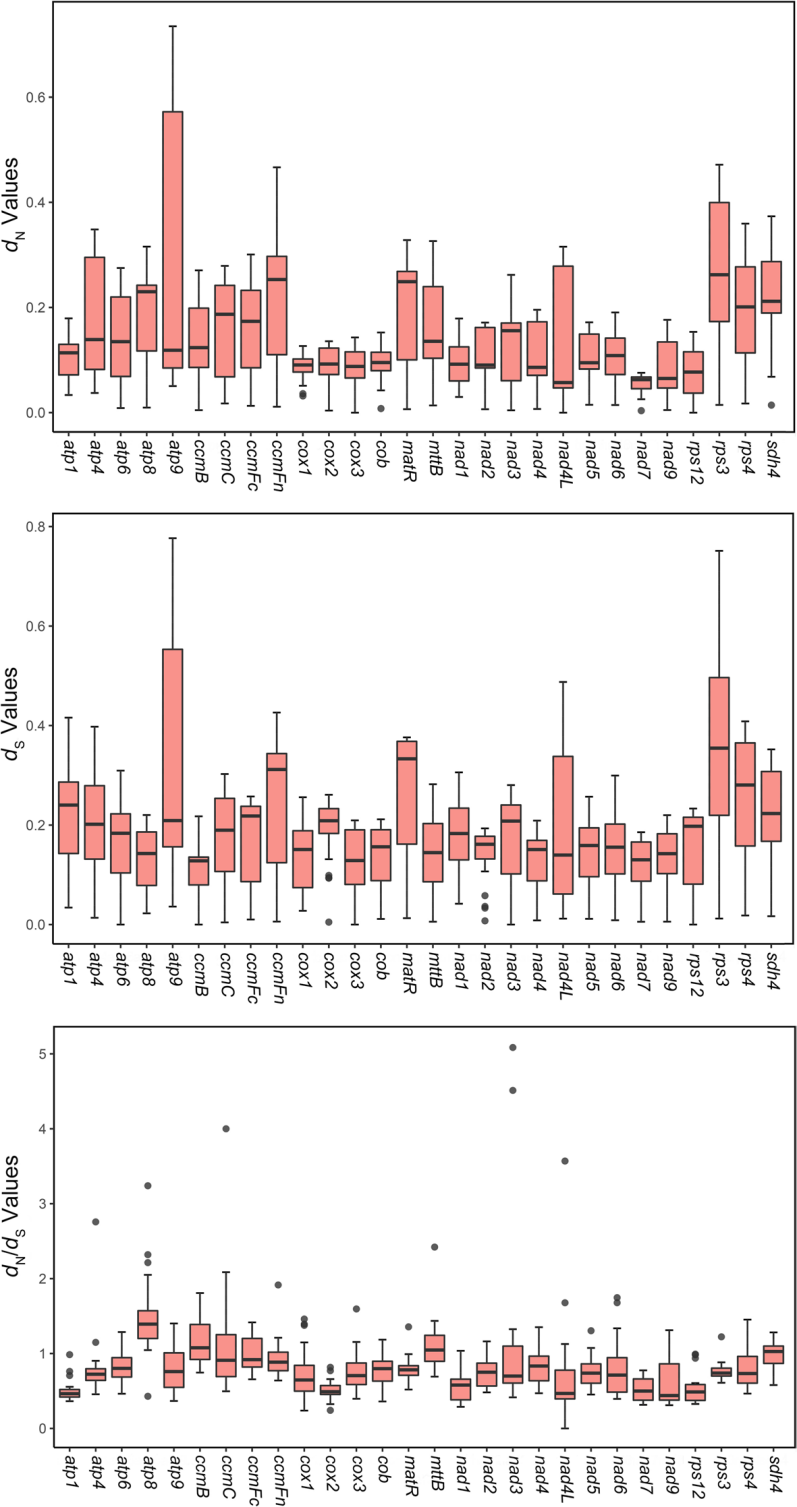
Boxplots of pairwise *d*_N_, *d*_S_ values and their ratio among 28 mitochondrial genes in the 7 gymnosperm mitogenomes

We extracted 28 shared conserved mitochondrial genes from 7 representative gymnosperms and calculated the nucleotide substitution rates at two levels (species and genes). The nucleotide substitution rate at the species level was calculated using a supermatrix concatenating 28 genes. The results showed that most *d*_N_/*d*_S_ values were less than 1.0, with the exception of *W. mirabilis* vs. *Pi. Taeda* and *Pl. orientalis* vs. *Th. sutchuenensis*, indicating that almost all gymnosperms were negatively selected (Additional file 1: Fig. S10, Additional file 2: Table S2. 29). At the gene level, the paired nucleotide substitution rates of these 28 genes were calculated respectively. The results indicated that most genes had *d*_N_/*d*_S_ values less than 1.0 (Fig. 6, Additional file 2: Table S2. 30), suggesting that they had undergone purifying selection, especially *atp1*, *cox2*, *nad7* and *rps12*, which were less than 1.0 in all species. In contrast, a few genes, including *atp8*, *ccmB*, *mttB* and *sdh4*, had *d*_N_/*d*_S_ values that were greater than 1.0 in most species, suggesting potential positive selection. The boxplot also revealed that *atp1, nad4L* and *nad9* had unusually low *d*_N_/*d*_S_ values, which may indicate that they have undergone intense purification and were well conserved in the mitogenome of gymnosperms (Fig. 7).

## Discussion

The National Center for Biotechnology Information (NCBI) released 7,943 chloroplast genomes and 470 mitochondrial genomes as of July 28, 2022. As an increasing number of mitogenomes are published, studies have found that the size of plant mitogenomes varies greatly, ranging from 66 kb to 11.7 Mb [12, 27]. Additionally, studies have revealed that plant mitogenomes differ from chloroplast genomes (always containing the conserved single loop structure) and should be presented in multiple sequence units to show the dynamic connection of the mitogenome [3, 5, 6, 28, 29]. The complete mitochondrial genome of *Th. sutchuenensis* was assembled and validated in this study, which consists of one circular contig and three linear contigs with overlapping regions among each other (Fig. 1, 2). The use of long reads is critical to achieving this complete assembly. The multipart mitogenome described in our study was also reported in other gymnosperms, such as *Pl. orientalis*, *Larix sibirica* and *Picea sitchensis* (Bong.) Carrière, which were composed of 2, 9 and 13 segments, respectively [4, 12, 13].

SSRs and dispersed repeats are important components of repeat sequences in the mitochondrial genome [5, 6, 8]. By comparing the repeat sequences of 7 gymnosperm mitogenomes, it is interesting to find that the number of SSRs in gymnosperms form a definite hierarchy and could well reflect the phylogenetic relationship. The number of SSRs could be divided into three groups, namely, *C. taitungensis* and *G. biloba* (both less than 200), *Pi. taeda* and *W. mirabilis* (500–700), and *Ta. cuspidata, Pl. orientalis* and *Th. Sutchuenensis* (more than 1,000), which may indicate that the size of the SSR sequence of gymnosperms has gradually increased during evolution (Additional file 1: Fig. S6A).

Previous studies have indicated that repeat sequences in the mitogenome are crucial for intermolecular recombination, especially long repeat sequences (more than 1 kb), which may cause high-frequency recombination, leading to isomerization of the genome into multiple major forms [11, 29, 30]. Our study revealed that both the number and length of long repeats of *Th. sutchuenensis* and *Pl. orientalis* were far more than those of the other 5 gymnosperms, and the longest repeat was also found in *Th. sutchuenensis*, with a length of more than 47 kb (Additional file 1: Fig. S7). This longest repeat was located in contig 3, whose two ends formed a complex connected structure with contig1 and contig2 (Fig. 1, 3; Additional file 1: Fig. S5). The accuracy of this connected structure has also been confirmed by our experiment. Therefore, it can be inferred that the recombination mediated by these abundant long repeats may help the mitogenome of *Th. sutchuenensis* to form a more complicated conformation than that of other gymnosperms [29–31].

The mitogenome of angiosperms has 29 conserved collinear gene clusters, according to earlier research [26]. Among the angiosperms selected in that study, the number of collinear gene clusters ranged from 7 to 23. However, compared to angiosperms, our study revealed that gymnosperms have a significantly lower number of collinear gene clusters, with *G. biloba* having the most, with 8 gene clusters, and *W. mirabilis* having the fewest, with just 2 (Additional file 1: Fig. S9) [8]. Only one collinear gene cluster, *nad3*-*rps12*, is shared by all of the selected gymnosperm species. Additionally, comparative research revealed that *Th. sutchuenensis* and *Pl. orientalis* have identical compositions of collinear gene clusters. These findings indicated that in gymnosperms, the orders of mitochondrial genes may only be conserved within families but vary greatly in different lineages of gymnosperms, which may be connected to frequent recombination caused by abundant repeat sequences in gymnosperm mitogenomes.

Previous studies on the tRNAs of gymnosperms were mostly based on software prediction, which may overestimate the number of tRNAs. BLAST based on sequences could more accurately reflect the composition of tRNAs. Thus, we integrated a tRNA database from previous studies and used it to identify potential tRNAs for 7 representative gymnosperms [26, 32, 33]. The analysis showed that extensive loss events of tRNA (mitochondrial origin) have occurred in the evolutionary history of gymnosperm mitogenomes, and only a few tRNAs were conserved in the evolutionary process (Fig. 5B). Surprisingly, our results indicated that tRNAs of chloroplast origin may have a potential functional complement. For example, *W. mirabilis* lost the *trnfM-CAU* of mitochondrial origin that was present in the other 6 gymnosperms, but it was found to harbour the only *trnfM-CAU* of chloroplast origin, which may play a complementary role in function.

The *d*_N_/*d*_S_ analysis in our study increased our comprehension of the mitochondrial gene evolution of gymnosperms. The analysis indicated that most genes of these 7 gymnosperms had *d*_N_/*d*_S_ values that were less than 1.0, which may imply that they were conservative and underwent purifying selection (Fig. 6). This inference was consistent with the conclusions of other studies on angiosperms [6, 34]. However, we also identified that four genes, *atp8*, *ccmB*, *mttB* and *sdh4*, may have undergone positive selection in the evolution of gymnosperms in most species. These four genes are involved in the biosynthesis of ATP synthase, ubiquinol cytochrome c reductase, transport membrane protein and succinate dehydrogenase, respectively [5, 7]. Based on previous studies, it is speculated that these four genes may have undergone positive selection under environmental stress to generate new functions to adapt to the new environment.

## Conclusion

In this study, we successfully assembled the mitochondrial genome of *Th. sutchuenensis* by using Nanopore and DNBSEQ reads, which is the second most complete mitogenome of Cupressaceae. The complex conformation of the mitogenome of *Th. sutchuenensis,* whose presence was validated by experiments and sequencing, was composed of three linear contigs and one small circular contig. The results of this study further proved the authenticity of the mitogenome's complex structure in seed plants. By comparing the mitogenomes of five different lineages of gymnosperms, we discovered that the number of SSRs may have increased during the evolutionary history of gymnosperms and that these abundant long repeats may contribute to the generation of the complex conformation of the mitogenome of *Th. sutchuenensis*. The analysis of the collinear block and the gene cluster revealed that the orders of mitochondrial genes of *Th. sutchuenensis* was not conserved, which indirectly proved that this mitogenome might have undergone a high level of recombination. In addition, the analysis of tRNA genes indicated that only a few tRNAs were conserved in the evolutionary process, and tRNAs of chloroplast origin may have potential complementary functions. The investigation of *Th. sutchuenensis* mitogenome in this study also offers a theoretical foundation for removing the reproductive barriers faced by this threatened species.

## Methods

### Sampling and genome sequencing

We collected young needles of *Th. sutchuenensis* from the ecological park of Southwest University and extracted total genomic DNA using the CTAB method [35]. Voucher specimens for this plant were collected and deposited in the herbarium of Southwest University, Chongqing, China, with accession number: 20210425TS-1. A DNA library with an insert size of 350 bp was constructed using the kit and sequenced using the DNBSEQ sequencing platform. Soapnuke (V 1.6.5) [36] was used to filter the raw data, which could remove low-quality reads and some reads with adapter contamination or PCR duplication. Finally, a total of 40 G of clean data was obtained.

The total genomic DNA of the identical plant sample used for DNBSEQ short-read sequencing was also subsequently used for Oxford Nanopore sequencing, which followed the standard protocol provided by Oxford Nanopore Technologies (ONT) company and included sample quality detection, library construction, library quality detection, library sequencing and other processes. In total, 15 Gb of sequence reads were obtained, and 13.71 Gb remained after filtering and qualification. The average read length of filtered reads was 8.16 kb (N50 = 17.69 kb), and the longest read was 733.08 kb.

### Mitogenome assembly and annotation

We first used Flye (version 2.9-b1774) [37] to assemble the draft mitogenome through Nanopore sequencing data. Here, all the raw data were used as input files, and the nuclear genome, mitochondrial genome and chloroplast genome were assembled. The minimum overlap was set to 2,000, and the other parameters were set to default. For the assembled contigs, we exported them into FASTA format and made a database by using makeblastdb. The conserved mitochondrial genes of *Ta. cuspidata* (MN593023) and *Pl. orientalis* (OL703044 and OL703045) were used as the query sequence, and the BLASTn program [38] was used to identify the assembly contigs containing mitochondrial genes. Finally, we imported the GFA format file generated by Flye in Bandage [25] software, the identified mitochondrial contigs were retained and the others were removed. These mitochondrial contigs had overlapping regions with each other, and formed a complex graph, which was considered the draft mitochondrial genome. Subsequently, we mapped these long reads to the draft genome to resolve repeat regions. In short, for each repeated region, the path supported by most long reads was the optimal path. The repeat resolved sequences represent the major conformation of the dynamically changing mitochondrial genome. Finally, four contigs were obtained and considered to be the hypothetical mitogenome of *Th. sutchuenensis* (Fig. 1). To obtain the final mitogenome sequence, we used both short-read data and long-read data to correct these four contigs with minimap2/miniasm [39, 40], racon (v1.4.20) and pilon (v1.23) [41, 42].

### Validating the assembled conformation

PCR experiments with specific primer pairs were used to amplify the connected regions of three contigs, which could verify the presence of this complex conformation. Four pairs of specific primers were designed for 4 connecting regions using Primer blast of NCBI, which are listed in Table S3.2 (Additional file 1: Fig. S11, Additional file 3: Table S3.2). PCRs were performed in a 50 μl mixture, including 10 μl buffer, 4 μl dNTPs, 1 μl hyPerFUsion™ High Fidelity DNA Polymerase, 2 μl total DNA, 2 μl each of the forwards and reverse primers, and 29 μl ddH_2_O. After an initial denaturation at 94 °C for 2 min, PCRs were performed for 35 cycles. Each cycle consisted of denaturation at 94 °C for 30 s, annealing at 55 °C for 30 s, and elongation at 72 °C for 1 min. The PCR products were sequenced by Sanger sequencing.

### Genome annotation

Geseq [43] was used to annotate the mitogenome. For the annotation of transfer RNA genes, we first built a local tRNA database based on many previous studies and then used four contigs as queries to blast potential tRNA genes [26, 32, 33]. After blast, sequences with an identity less than 85% or a length difference (compared with the target sequence) greater than 10 bp were removed, which may be considered incomplete. Second, tRNAscan SE [44] was used to reconfirm the accuracy of the blasting results. Finally, each annotation error of the mitochondrial genome was manually modified and corrected by Apollo [45]. The mitogenome map was drawn with OGDRAW [46]. The annotated mitogenome of *Th. sutchuenensis* was submitted to NCBI under accession numbers ON603305-ON603308.

### Identification of repeat elements

The repeat sequences of each contig were annotated separately. Then, the long repeats (> 500 bp) were annotated for the whole mitogenome of *Th. sutchuenensis*. The online website MISA (https://webblast.ipk-gatersleben.de/misa/) was used to identify SSRs, with minimum repetition numbers of mono-, di-, tri-, tetra-, penta-, and hexanucleotides was 10, 5, 4, 3, 3, and 3, respectively [47]. Dispersed repeats were identified by using REPuter (https://bibiserv.cebitec.uni-bielefeld.de/reputer), with the hamming distance = 3, maximum computed repeats = 5,000, and minimal repeat size = 30 [48]. We also used Tandem Repeats Finder (https://tandem.bu.edu/trf/trf.html) to identify tandem repeats with default settings [49]. Additionally, six mitochondrial genomes of representative gymnosperms were downloaded from NCBI, including *C. taitungensis* (AP009381), *G. biloba* (KM672373), *W. mirabilis* (KT313400), *Pi. taeda* (MF991879), *Ta. cuspidata* (MN593023) and *Pl. orientalis* (OL703044, OL703045). These six mitogenomes, together with *Th. sutchuenensis*, were analysed for repeat sequences.

### Identification of MTPTs and synteny analysis

To identify the potential homologous sequences that may be transferred between the plastid (MH784400.1) and mitogenome of *Th. sutchuenensis*, we used BLASTn [38] to compare the two organelles, with the following parameter settings: E-value = 1e-5, NumofHits = 50,000, NumofAligns = 25,000. TBtools [50] was used for the visualization of the blast results. The identified homologous sequences were also extracted and then annotated with GeSeq. In addition, MCscan [51] was used to plot multiple synthetic plots of *Th. sutchuenensis* and six other gymnosperms based on sequence similarity. Moreover, we searched for gene clusters common to gymnosperms by simple visual inspection to evaluate the conservation of gene orders for the above seven gymnosperms.

### Estimation of nucleotide substitution rates

We used Phylosuite (V1.2.1) [52] to identify and extract the homologous mitochondrial genes in these six representative gymnosperms plus *Th. sutchuenensis*. The corresponding nucleotide sequences were aligned and concatenated by using Mafft (v7.450) [53] and Phylosuite, respectively. The yn00 module in PAML (v4.9) [54] was used to estimate the pairwise nucleotide substitution rates, including the nonsynonymous substitution rate (*d*_N_), synonymous substitution rate (*d*_S_), and ratio of *d*_N_ to *d*_S_. Single-gene matrix and concatenated matrix were used as input files to calculate the nucleotide substitution rates at the gene level and species level, respectively. TBtools and R-package (ggplot) were used to draw boxplots and heatmaps for pairwise *d*_N_/*d*_S_ values [50, 55].

## Supplementary Information

**Additional file 1: Figure S1. **Gel electrophoresis imagefor the PCR products. M, marker; 1-6, the ID of the duplicated biologicalsamples. The expected lengths of each fragment are shown at the bottom of thegel. **Figure S2. **Sanger sequencing results. Panels **A**, **B**, **C** and **D** are the alignment of corresponding genomic regions (the first row) with the PCR products (all rows except the first row). A: the connection region 1 between contig 1 and contig 3; B: the connection region 2 between contig 1 and contig 3, C: the connection region between contig 1 and contig 2; D the connection region between contig 2 and contig 3. **Figure S3. **The histogram of simple sequence repeats (SSRs) identified in the 4 contigs of *Thuja sutchuenensis*. **Figure S4.** The histogram of dispersed repeats identified in the 4 contigs of *Thuja sutchuenensis. ***Figure S5. **The distribution of long repeats (>500 bp) in the whole mitogenome of *Thuja sutchuenensis. ***Figure S6.** The histogram of simple sequence repeats (SSRs) and dispersed repeats identified in the 7 gymnosperm mitogenomes. A and B shows the comparison of SSRs and dispersed repeats among the 7 gymnosperm mitogenomes, respectively. **Figure S7.** The histogram of long repeats identified in the 7 gymnosperm mitogenomes. **Figure S8.** Homologous fragments between the chloroplast and mitochondrial genome of *Thuja sutchuenensis*. The red, blue and green fragments represent homologous fragments with the percent of identity more than 90, more than 80 but less than 90, less than 80, respectively. **Figure S9. **The distribution of mitochondrial gene clusters in 7 gymnosperms. **Figure S10. **Heatmap of pairwise *d*_N_/*d*_S_ratios between each pair of sequences in the multigene nucleotide alignment. **Figure S11. **Distribution diagram of 4 pairs of specific primers.

**Additional file 2: Table S2. **1 The simple sequence repeats (SSRs) identified in contig 1 of *Thuja sutchuenensis*. 2 The simple sequence repeats (SSRs) identified in contig 2 of *Thuja* *sutchuenensis*. 3 The simple sequence repeats (SSRs) identified in contig 3 of *Thuja sutchuenensis*. 4 The simple sequence repeats (SSRs) identified in contig 4 of *Thuja sutchuenensis*. 5 The dispersed repeats identified in contig 1 of *Thuja sutchuenensis*. 6 The dispersed repeats identified in contig 2 of *Thuja sutchuenensis*. 7 The dispersed repeats identified in contig 3 of *Thuja sutchuenensis*. 8 The dispersed repeats identified in contig 4 of *Thuja sutchuenensis*. 9 The tandem repeats identified in contig 1 of *Thuja sutchuenensis*. 10 The tandem repeats identified in contig 2 of *Thuja sutchuenensis*. 11 The tandem repeats identified in contig 3 of *Thuja sutchuenensis*. 12 The tandem repeats identified in contig 4 of *Thuja sutchuenensis*. 13 The long repeats  (>500 bp) identified in the whole mitogenome of *Thuja sutchuenensis*. 14 The simple sequence repeats (SSRs) identified in the mitogenome of *Cycas taitungensis*. 15 The simple sequence repeats (SSRs) identified in the mitogenome of *Ginkgo biloba*. 16 The simple sequence repeats (SSRs) identified in the mitogenome of *Pinus taeda*. 17 The simple sequence repeats (SSRs) identified in the mitogenome of *Welwitschia mirabilis*. 18 The simple sequence repeats (SSRs) identified in the mitogenome of *Taxus cuspidata*. 19 The simple sequence repeats (SSRs) identified in contig 1 of *Platycladus orientalis*. 20 The simple sequence repeats (SSRs) identified in contig 2 of *Platycladus orientalis*. 21 The dispersed repeats identified in the mitogenome of *Cycas taitungensis*. 22 The dispersed repeats identified in the mitogenome of *Ginkgo biloba*. 23 The dispersed repeats identified in the mitogenome of *Pinus taeda*. 24 The dispersed repeats identified in the mitogenome of *Welwitschia mirabilis*. 25 The dispersed repeats identified in the mitogenome of *Taxus cuspidata*. 26 The dispersed repeats identified in contig 1 of *Platycladus orientalis*. 27 The dispersed repeats identified in contig 2 of *Platycladus orientalis*. 28 The long repeats (>500 bp) identified in the whole mitogenome of *Platycladus orientalis*. 29 Pairwise *d*_N_/*d*_S_ ratios between each pair of sequences in the multigene nucleotide alignment of 7 gymnosperms plants. 30 Pairwise *d*_N_/*d*_S_ ratios in different mitochondrial genes of 7 gymnosperms plants.

**Additional file 3: Table S3. **1 General features of seven gymnosperm mitogenomes. **Table S3.** 2 Primers used in this study. 

## Data Availability

The sequencing data for the DNBSEQ and Nanopore platforms and the mitogenome sequences have been deposited in NCBI (https://www.ncbi.nlm.nih.gov/) with accession number PRJNA872324, SRX17374268, SRX17299360 (https://www.ncbi.nlm.nih.gov/bioproject/PRJNA872324) and ON603305-ON603308.
