# Supplementary material for: Complete mitochondrial genome of Thuja sutchuenensis and its implications on evolutionary analysis of complex mitogenome architecture in Cupressaceae
Source: BMC Plant Biol. 2023 Feb 7;23:84. doi: 10.1186/s12870-023-04054-9 (PMC9903464; doi:10.1186/s12870-023-04054-9)

**Additional File 1. Supplementary Figures**

**(Figures S1-S11)**

**Complete mitochondrial genome of *Thuja sutchuenensis* and its implications on evolutionary analysis of complex mitogenome architecture in Cupressaceae**

**Figure S1.** Gel electrophoresis image for the PCR products. M, marker; 1-6, the ID of the duplicated biological samples. The expected lengths of each fragment are shown at the bottom of the gel.


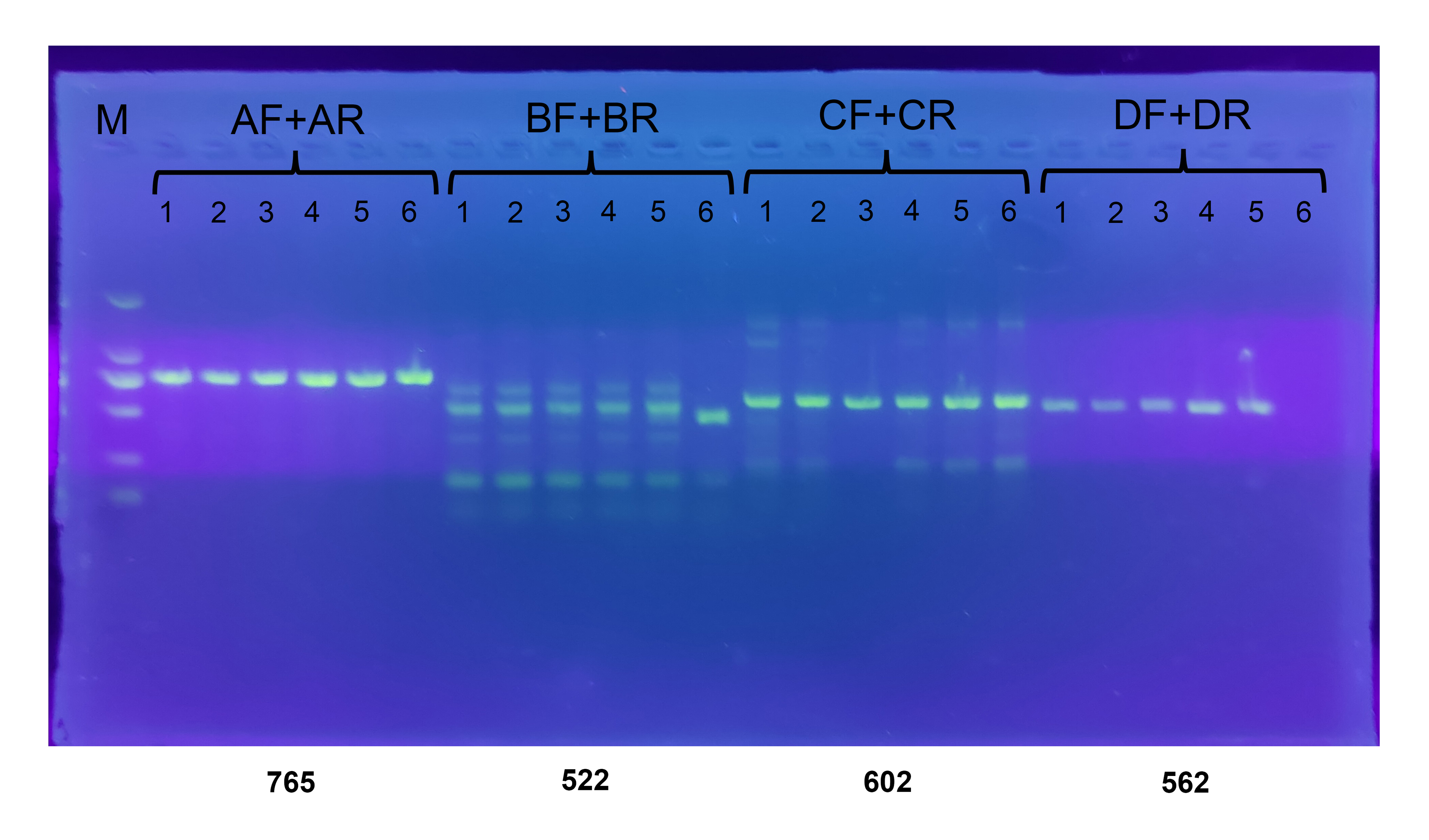


**Figure S2.** Sanger sequencing results. Panels A, B, C and D are the alignment of corresponding genomic regions with the PCR products. A: the connection region 1 between contig 1 and contig 3; B: the connection region 2 between contig 1 and contig 3, C: the connection region between contig 1 and contig 2; D the connection region between contig 2 and contig 3.


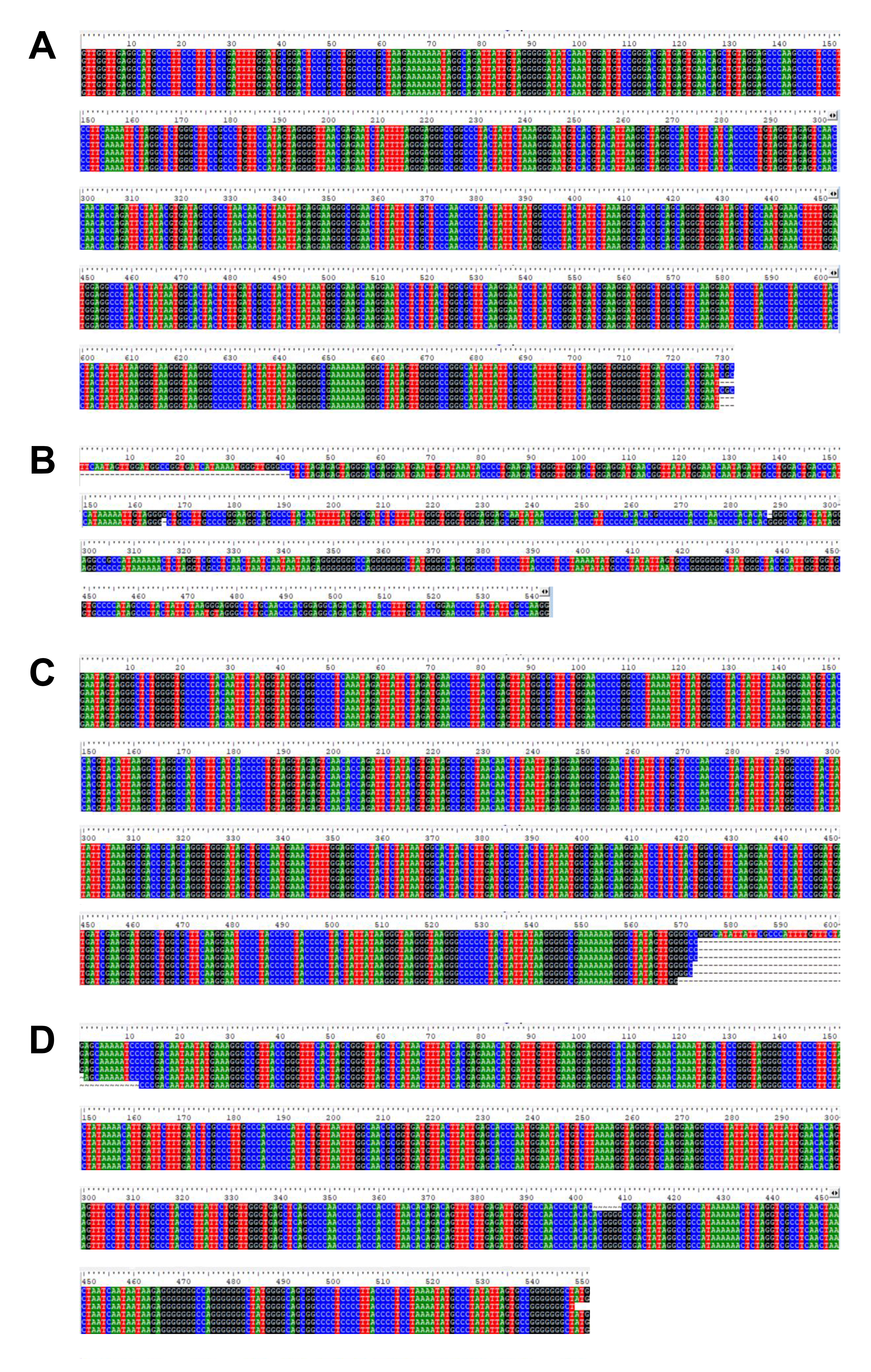


**Figure S3.** The histogram of simple sequence repeats (SSRs) identified in the 4 contigs of *Thuja sutchuenensis*.

**
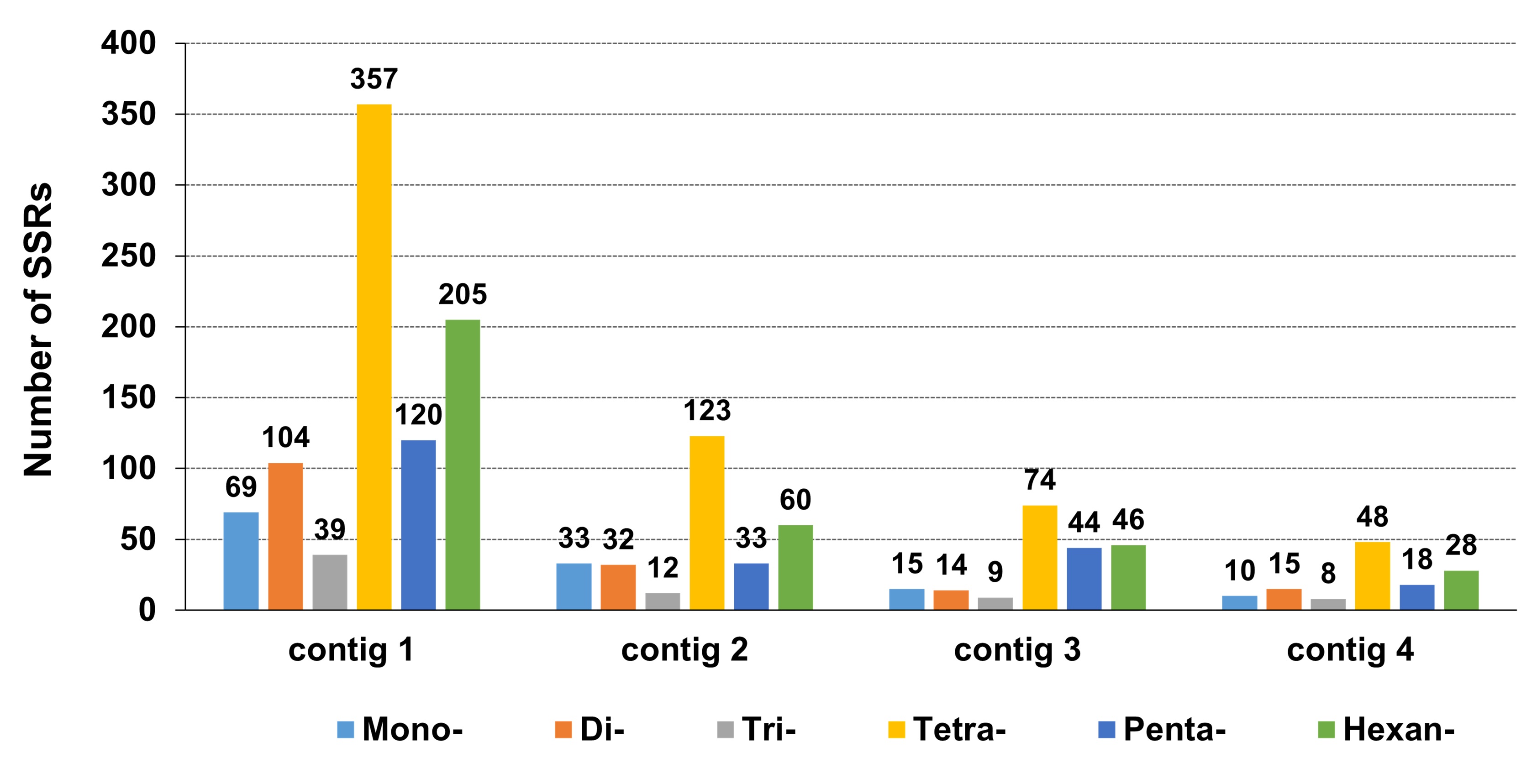
**

**Figure S4.** The histogram of dispersed repeats identified in the 4 contigs of *Thuja sutchuenensis.*


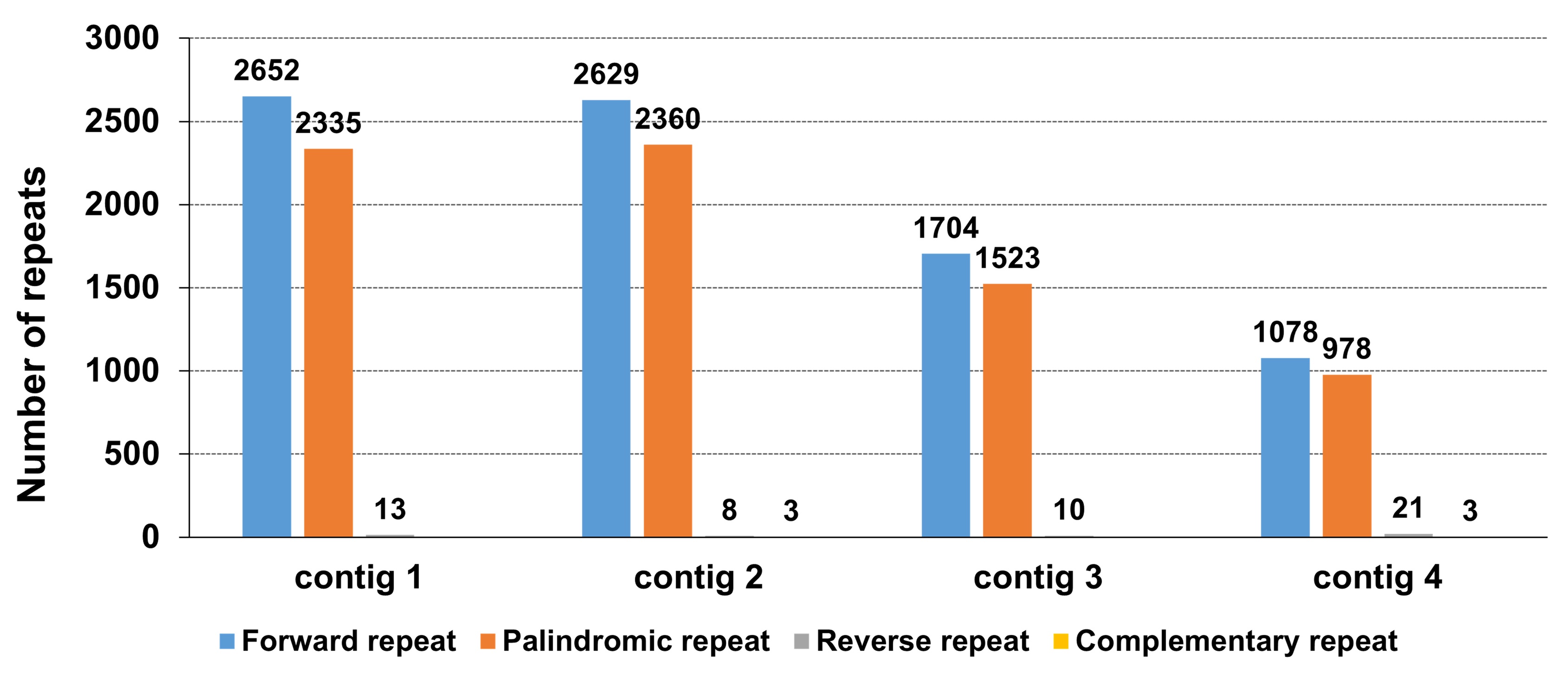


**Figure S5.** The distribution of long repeats (>500 bp) in the whole mitogenome of *Thuja sutchuenensis*.


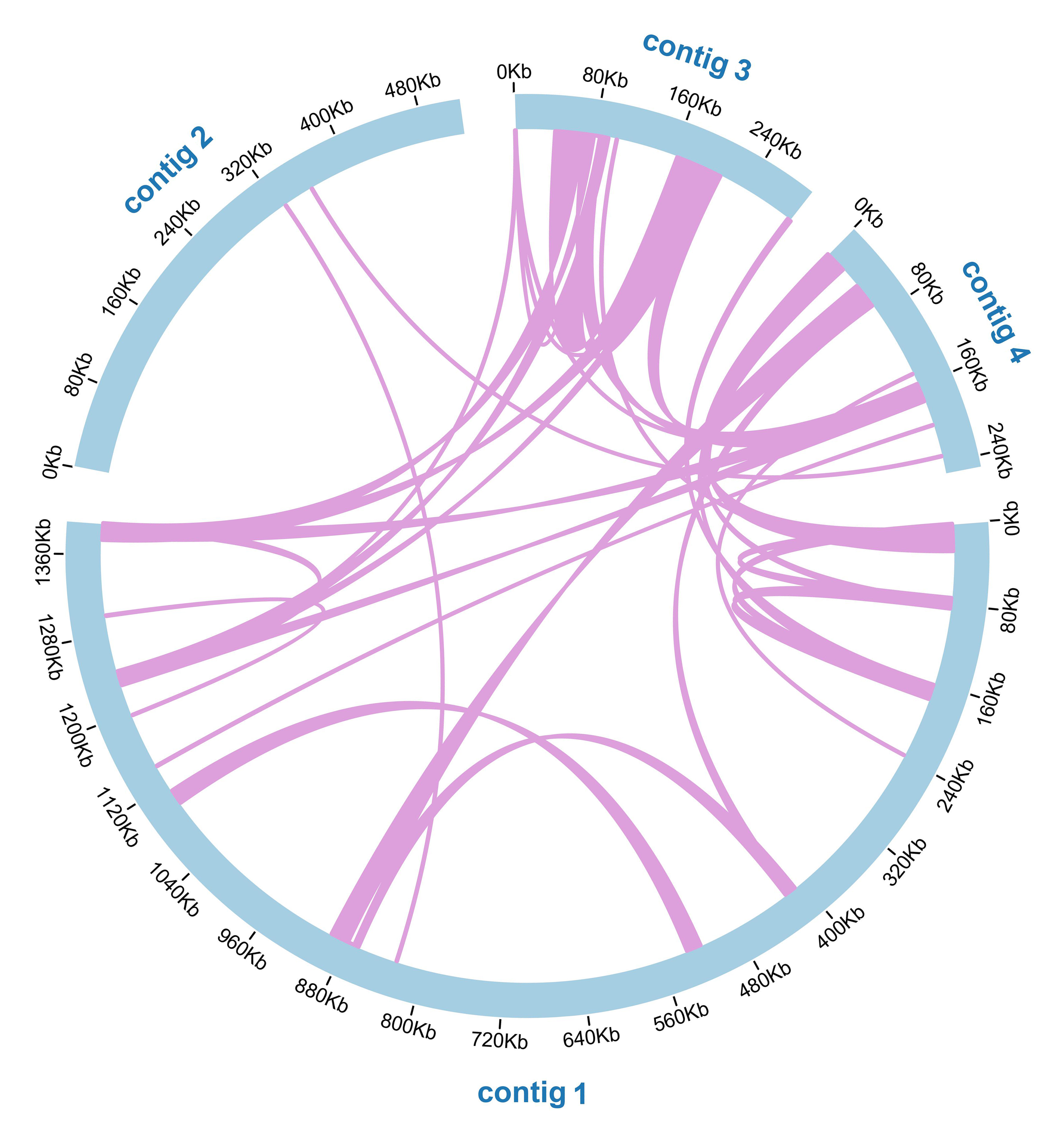


**Figure S6.** The histogram of simple sequence repeats (SSRs) and dispersed repeats identified in the 7 gymnosperm mitogenomes. A and B shows the comparison of SSRs and dispersed repeats among the 7 gymnosperm mitogenomes, respectively.


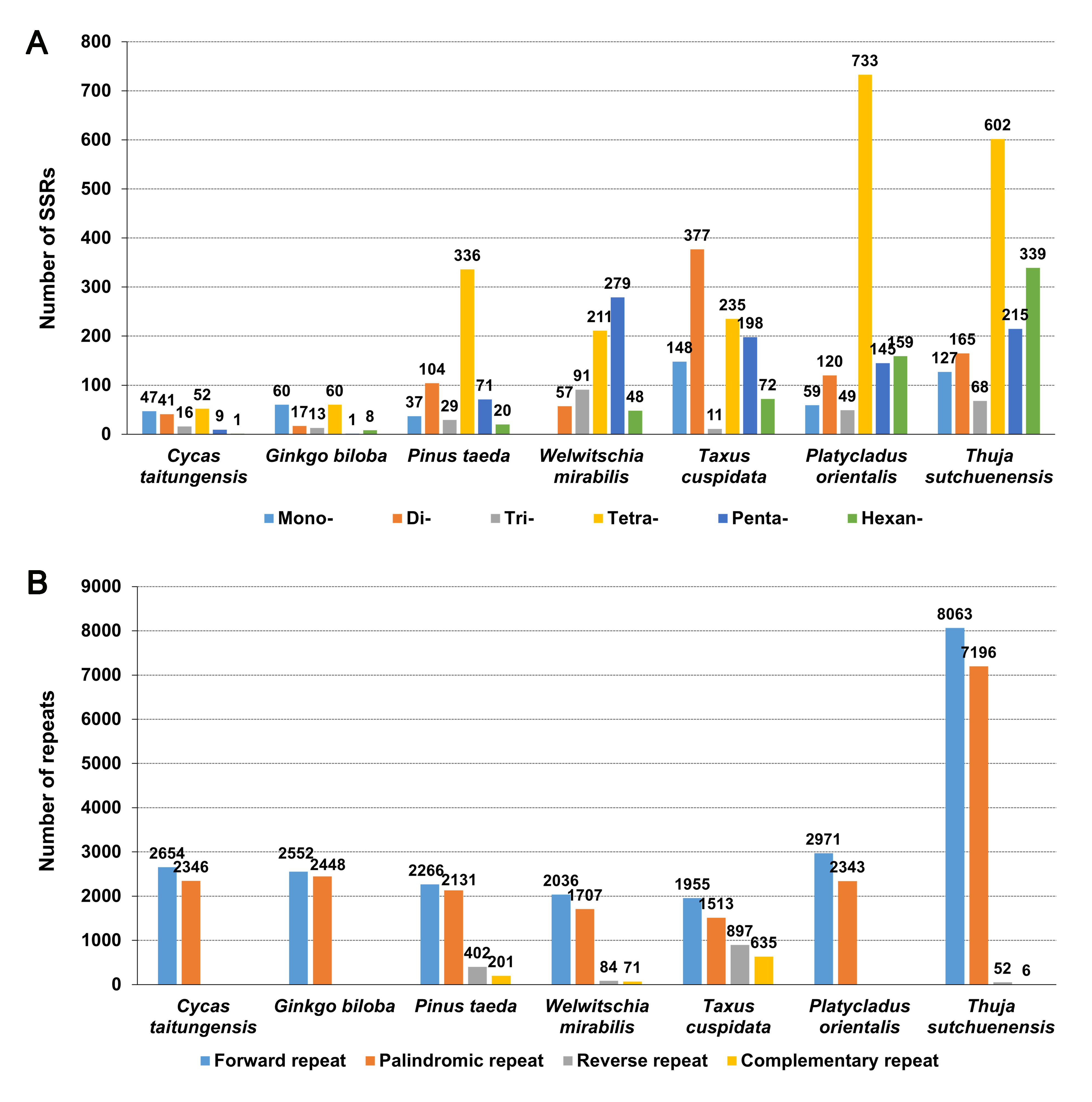


**Figure S7.** The histogram of long repeats identified in the 7 gymnosperm mitogenomes.


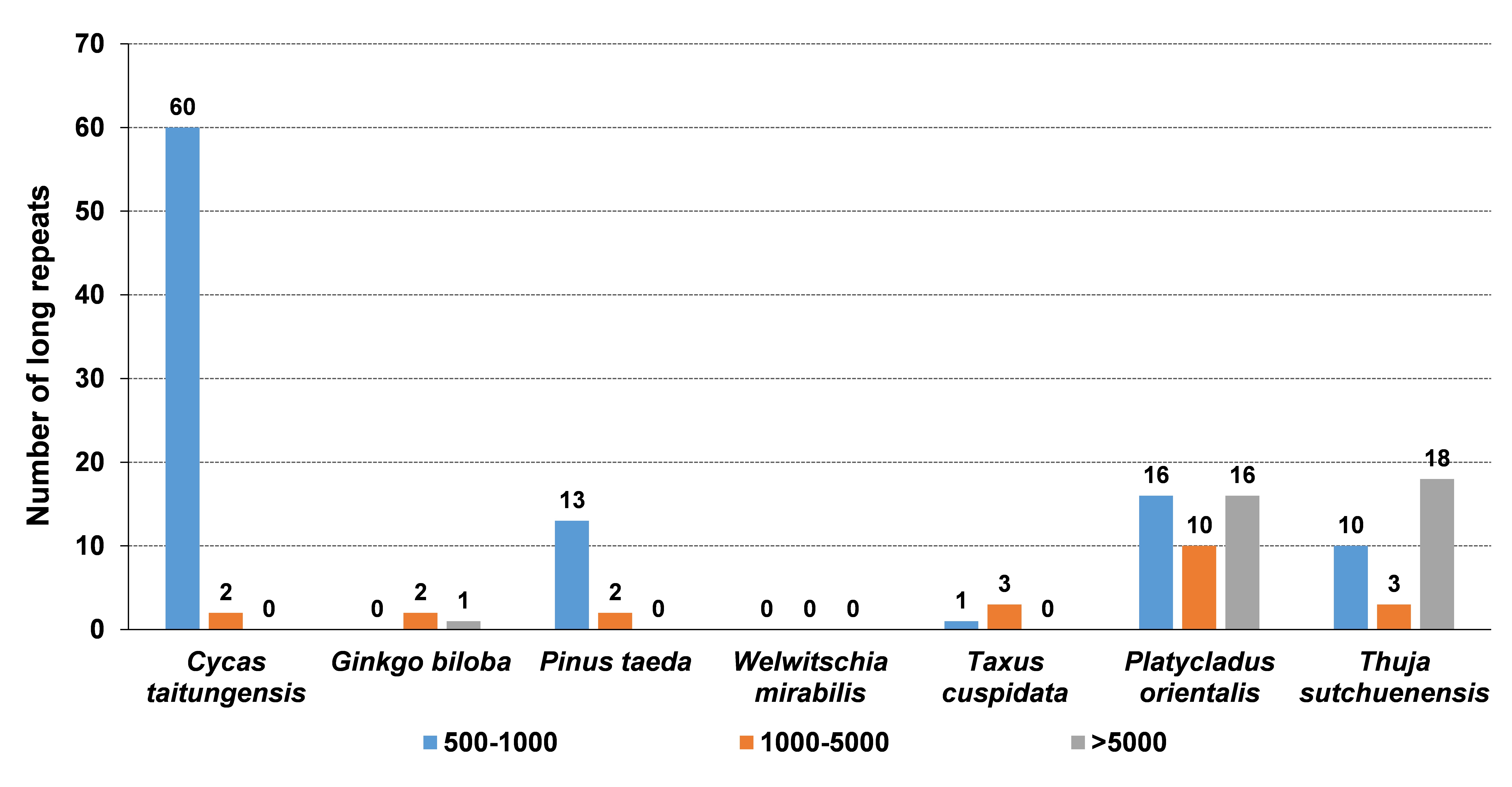


**Figure S8.** Homologous fragments between the chloroplast and mitochondrial genome of *Thuja sutchuenensis*. The red, blue and green fragments represent homologous fragments with the percent of identity more than 90, more than 80 but less than 80, less than 80, respectively.


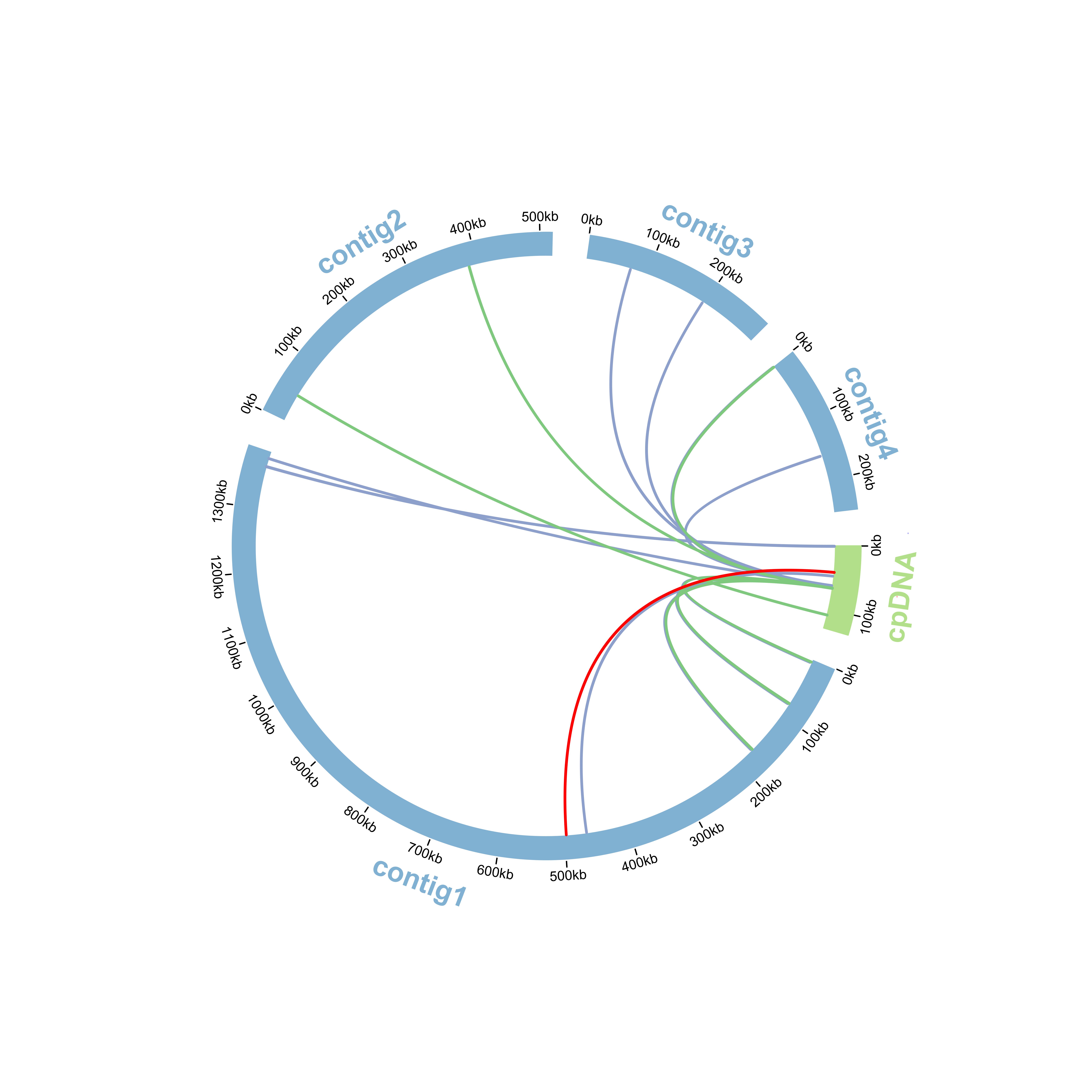


**Figure S9.** The distribution of mitochondrial gene clusters in 7 gymnosperms.


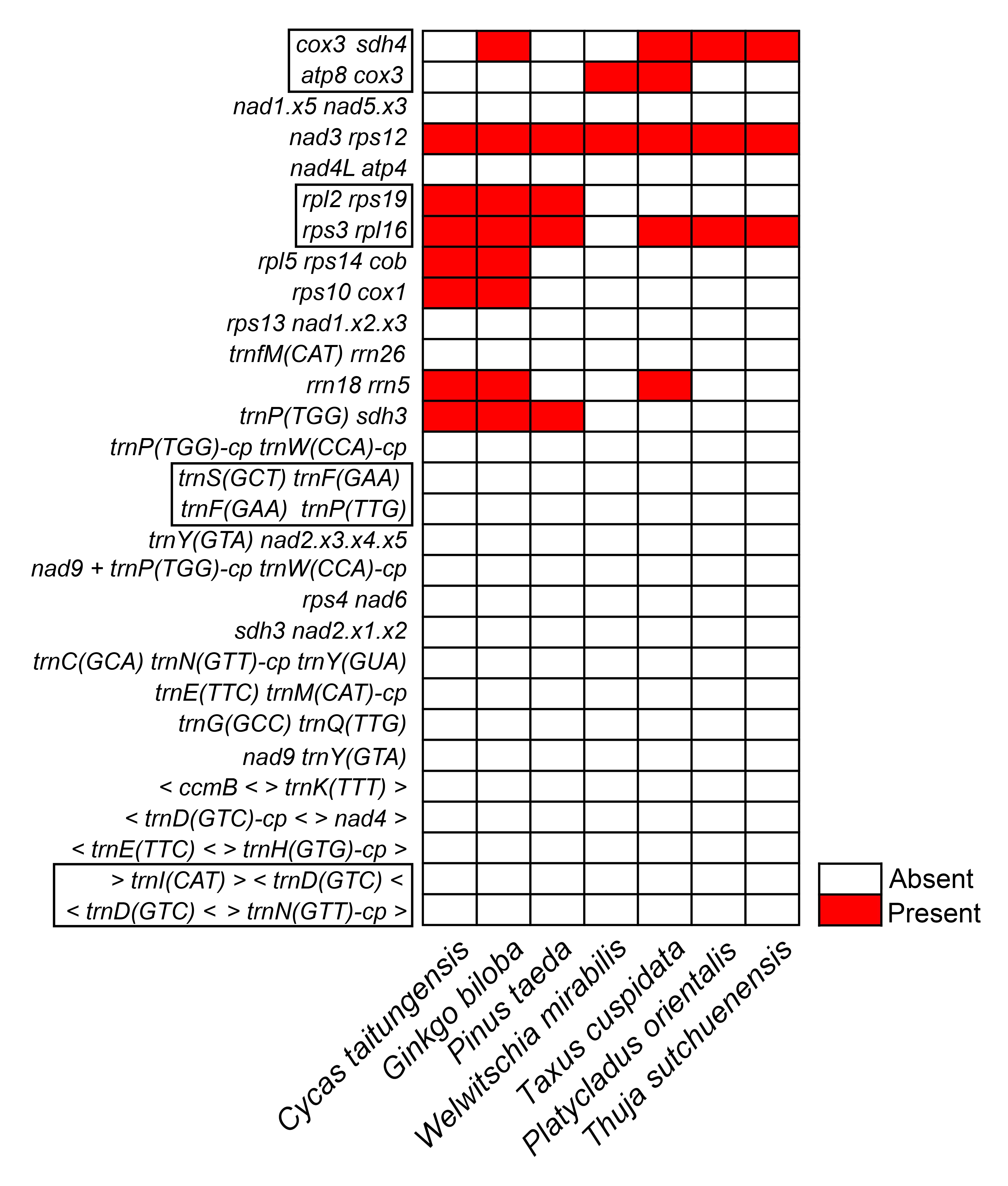


**Figure S10.** Heatmap of pairwise *d*_N_/*d*_S_ ratios between each pair of sequences in the multigene nucleotide alignment.


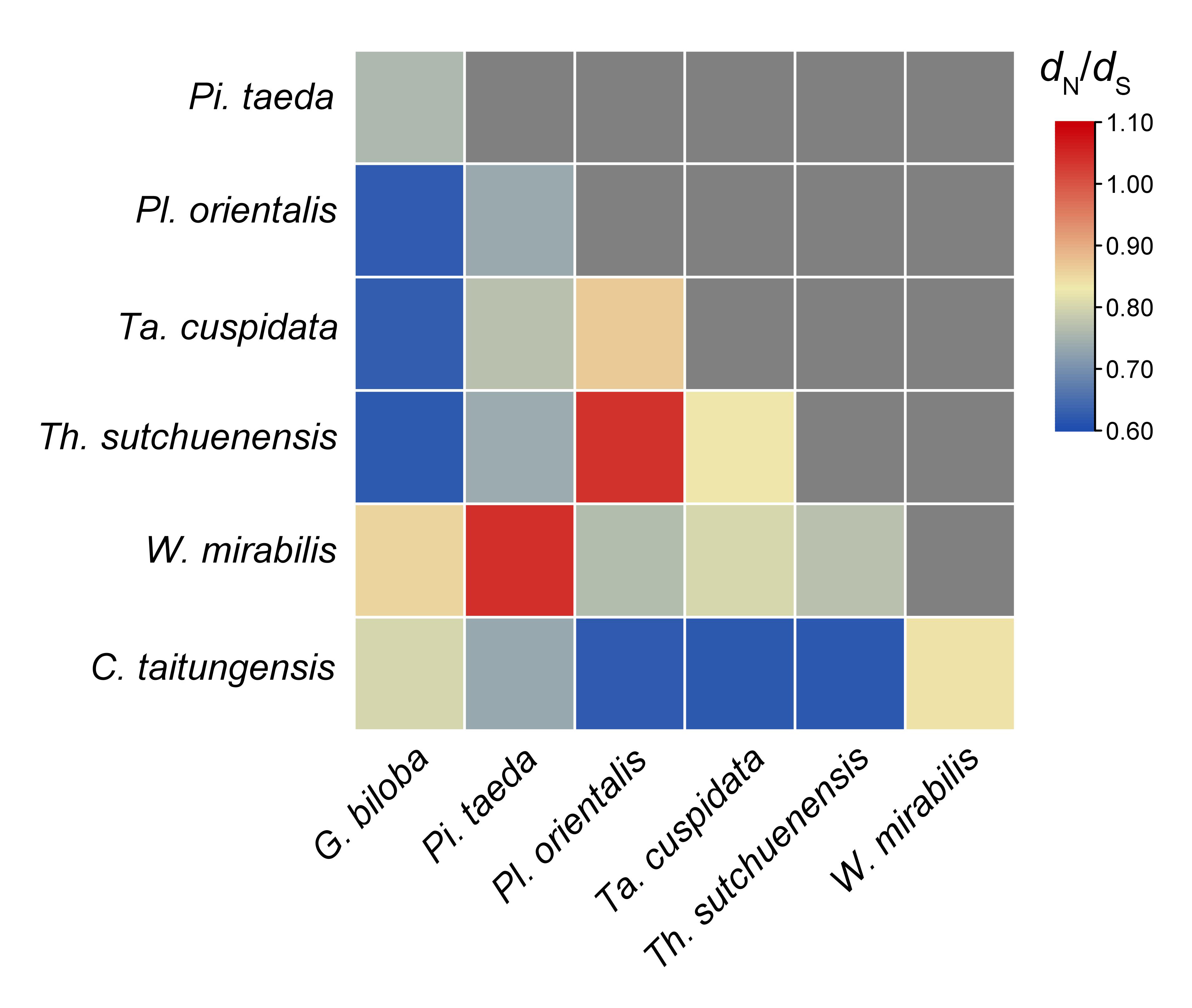


**Figure S11** Distribution diagram of 4 pairs of specific primers.


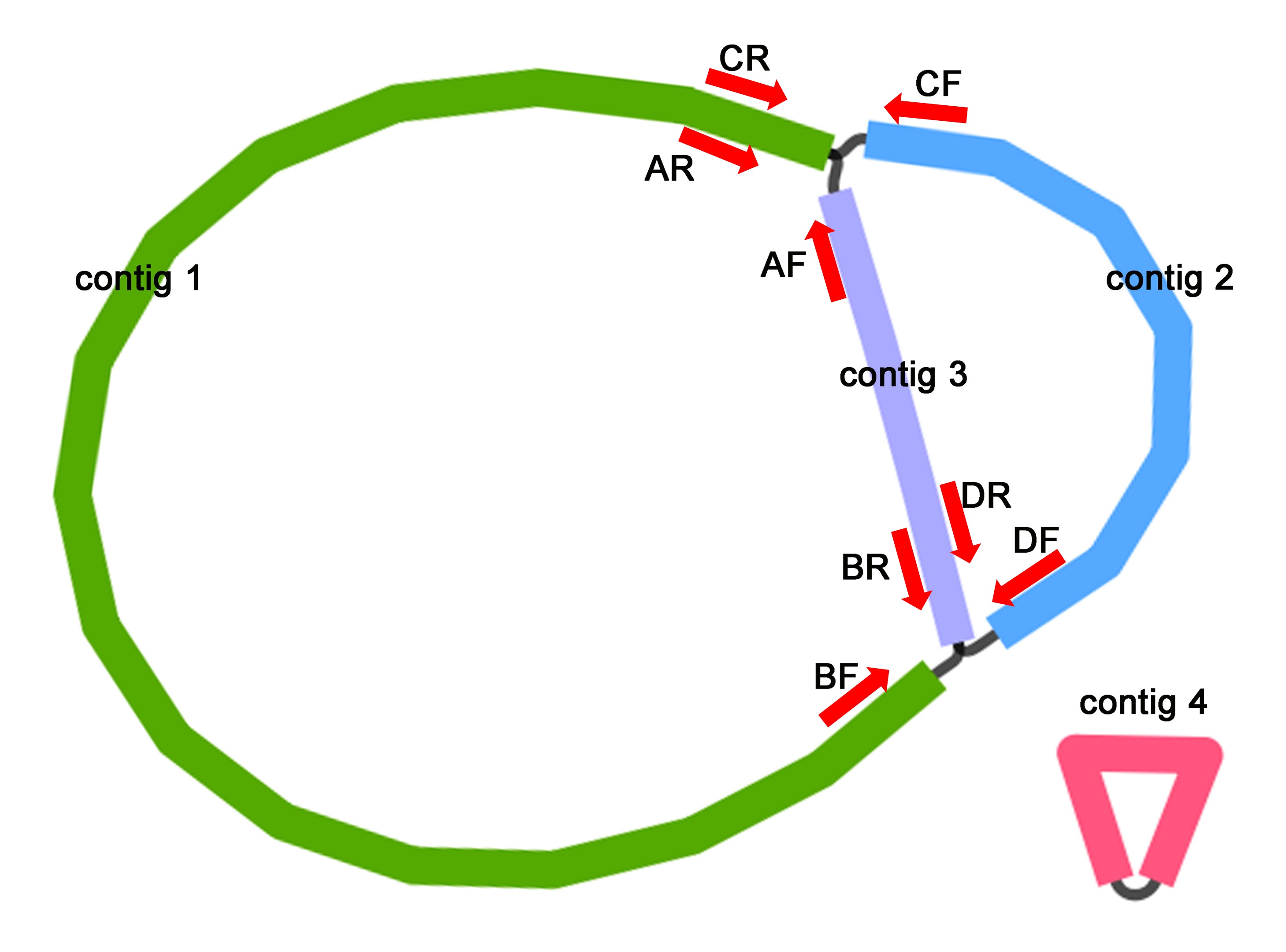

Supplement: Supplementary file 1 — Additional file 1: Figure S1. Gel electrophoresis imagefor the PCR products. M, marker; 1-6, the ID of the duplicated biologicalsamples. The expected lengths of each fragment are shown at the bottom of thegel. Figure S2. Sanger sequencing results. Panels A, B, C and D are the alignment of corresponding genomic regions (the first row) with the PCR products (all rows except the first row). A: the connection region 1 between contig 1 and contig 3; B: the connection region 2 between contig 1 and contig 3, C: the connection region between contig 1 and contig 2; D the connection region between contig 2 and contig 3. Figure S3. The histogram of simple sequence repeats (SSRs) identified in the 4 contigs of Thuja sutchuenensis. Figure S4. The histogram of dispersed repeats identified in the 4 contigs of Thuja sutchuenensis. Figure S5. The distribution of long repeats (>500 bp) in the whole mitogenome of Thuja sutchuenensis. Figure S6. The histogram of simple sequence repeats (SSRs) and dispersed repeats identified in the 7 gymnosperm mitogenomes. A and B shows the comparison of SSRs and dispersed repeats among the 7 gymnosperm mitogenomes, respectively. Figure S7. The histogram of long repeats identified in the 7 gymnosperm mitogenomes. Figure S8. Homologous fragments between the chloroplast and mitochondrial genome of Thuja sutchuenensis. The red, blue and green fragments represent homologous fragments with the percent of identity more than 90, more than 80 but less than 90, less than 80, respectively. Figure S9. The distribution of mitochondrial gene clusters in 7 gymnosperms. Figure S10. Heatmap of pairwise dN/dSratios between each pair of sequences in the multigene nucleotide alignment. Figure S11. Distribution diagram of 4 pairs of specific primers. [file 12870_2023_4054_MOESM1_ESM.doc]
