# Supplementary material for: Complete mitochondrial genome of Thuja sutchuenensis and its implications on evolutionary analysis of complex mitogenome architecture in Cupressaceae
Source: BMC Plant Biol. 2023 Feb 7;23:84. doi: 10.1186/s12870-023-04054-9 (PMC9903464; doi:10.1186/s12870-023-04054-9)
Supplement: Supplementary file 3 — Additional file 3: Table S3. 1 General features of seven gymnosperm mitogenomes. Table S3. 2 Primers used in this study. [file 12870_2023_4054_MOESM3_ESM.doc]

**Additional File 3. Supplementary Tables**

**(Tables S3.1- Tables S3.2)**

**Complete mitochondrial genome of *Thuja sutchuenensis* and its implications on evolutionary analysis of complex mitogenome architecture in Cupressaceae**

**Table S3.1.** General features of seven gymnosperm mitogenomes.

|  | ***Cycas*** ***taitungensis*** | ***Ginkgo***  ***biloba*** | ***Pinus***  ***taeda*** | ***Welwitschia***  ***mirabilis*** | ***Taxus***  ***cuspidata*** | ***Platycladus***  ***orientalis*** | ***Thuja***  ***sutchuenensis*** |
| --- | --- | --- | --- | --- | --- | --- | --- |
| **Accession** | AP009381 | KM672373 | MF991879 | KT313400 | MN593023 | OL703044-  OL703045 | ON603305-  ON603308 |
| **Size (bp)** | 414,903 | 346,544 | 1,191,054 | 978,846 | 468,924 | 2,624,300 | 2,455,856 |
| **GC%** | 46.9 | 50.4 | 47 | 53 | 50.39 | 50.6 | 49.38 |
| **Protein coding** | 41 | 41 | 41 | 29 | 32 | 32 | 32 |
| **Introns** | 26 | 25 | 26 | 10 | 15 | 15 | 14 |

Note: this table was modified from Kan et al. (2020).

**Table S3.2.** Primers used in this study.

| **Primer name** | **Primer sequence** |
| --- | --- |
| AF | 5’-CGGGGGAGTAGGAAGCTAGT-3’ |
| AR | 5’-CTATTCTCAAGGGCGGCGAT-3’ |
| BF | 5’-AATAGTTGGATGGCCGGTGA-3’ |
| BR | 5’-CCTTGGCGAATAGTAGGGGT-3’ |
| CF | 5’-CCACAACGGCCCCTTAGAAT-3’ |
| CR | 5’-GAATAATATGCCCGGCCCCA-3’ |
| DF | 5’-TGAGCAAAAATCCCCCGACA-3’ |
| DR | 5’-CCACCAATGCGTAGCCCATA-3’ |
